# Evaluating the effect of innovative motivation and supervision approaches on community health worker performance and retention in Uganda and Mozambique: study protocol for a randomised controlled trial

**DOI:** 10.1186/s13063-015-0657-6

**Published:** 2015-04-12

**Authors:** Karin Källander, Daniel Strachan, Seyi Soremekun, Zelee Hill, Raghu Lingam, James Tibenderana, Frida Kasteng, Anna Vassall, Sylvia Meek, Betty Kirkwood

**Affiliations:** Malaria Consortium, Development House, 56-64 Leonard Street, London, EC2A 4LT UK; Department of Public Health Sciences, Tomtebodavägen 18A, Karolinska Institutet, 17177 Stockholm, Sweden; Department of Epidemiology and Biostatistics, School of Public Health, Makerere University College of Health Science, PO box 8045, Kampala, Uganda; Institute for Global Health, University College London, 30 Guilford Street, London, WC1N 1EH UK; Department of Population Health, London School of Hygiene and Tropical Medicine, London, UK; Malaria Consortium Africa, Plot 25 Upper Naguru East Road, Kampala, Uganda; Department of Global Health and Development, London School of Hygiene and Tropical Medicine, Keppel Street, London, WC1E 7HT UK

**Keywords:** community health worker, integrated community case management, malaria, pneumonia, diarrhoea

## Abstract

**Background:**

If trained, equipped and utilised, community health workers (CHWs) delivering integrated community case management for sick children can potentially reduce child deaths by 60%. However, it is essential to maintain CHW motivation and performance. The inSCALE project aims to evaluate, using a cluster randomised controlled trial, the effect of interventions to increase CHW supervision and performance on the coverage of appropriate treatment for children with diarrhoea, pneumonia and malaria.

**Methods/Design:**

Participatory methods were used to identify best practices and innovative solutions. Quantitative community based baseline surveys were conducted to allow restricted randomisation of clusters into intervention and control arms. Individual informed consent was obtained from all respondents. Following formative research and stakeholder consultations, two intervention packages were developed in Uganda and one in Mozambique. In Uganda, approximately 3,500 CHWs in 39 clusters were randomised into a mobile health (mHealth) arm, a participatory community engagement arm and a control arm. In Mozambique, 275 CHWs in 12 clusters were randomised into a mHealth arm and a control arm. The mHealth interventions encompass three components: 1) free phone communication between users; 2) data submission using phones with automated feedback, messages to supervisors for targeted supervision, and online data access for district statisticians; and 3) motivational messages. The community engagement arm in Uganda established village health clubs seeking to 1) improve the status and standing of CHWs, 2) increase demand for health services and 3) communicate that CHWs’ work is important. Process evaluation was conducted after 10 months and end-line surveys will establish impact after 12 months in Uganda and 18 months in Mozambique. Main outcomes include proportion of sick children appropriately treated, CHW performance and motivation, and cost effectiveness of interventions.

**Discussion:**

Study strengths include a user-centred design to the innovations, while weaknesses include the lack of a robust measurement of coverage of appropriate treatment. Evidence of cost-effective innovations that increase motivation and performance of CHWs can potentially increase sustainable coverage of iCCM at scale.

**Trial registration:**

(identifier NCT01972321) on 22 April 22 2013

## Background

During the last decade, child mortality has been reduced significantly in a number of African countries. However, preventable illnesses such as diarrhoea, pneumonia and malaria are still claiming almost 2 million lives in newborns, infants and children under five years of age [1]. Scaling up interventions that increase access to timely and appropriate treatment at the community level could prevent more than 60% of these deaths [2]. As a way of increasing access to treatment for sick children, several African countries are investing in community health workers (CHWs) as a cost-effective way of extending health services to people living beyond the reach of the health facilities. Integrated community case management (iCCM) is a delivery strategy that utilises CHWs to diagnose and treat multiple conditions, most commonly pneumonia, diarrhoea and malaria, in children under five [3]. If properly trained, equipped and utilised, CHWs have the potential to reduce child deaths substantially by increasing access to timely, appropriate and affordable treatment for poor and rural populations [4].

CHW programmes have been faced with the challenge to scale up with high CHW attrition rates and substandard care quality. This has largely been due to problems with poor planning; fragmented and disease-specific training; tenuous linkage to the health system; poor coordination, supervision and support; and under-recognition of CHWs’ contribution to the health system [5,6]. If CHW programmes are to reach their potential, there is an urgent need for strategies that improve performance, motivation and retention of CHWs [7-9]. This paper presents a protocol for a cluster randomised controlled trial (cRCT) conducted under the inSCALE project (Innovations at Scale for Community Access and Lasting Effects). inSCALE aims to enhance the motivation and performance of CHWs in order to ultimately increase the coverage of children who receive appropriate treatment for diarrhoea, pneumonia and malaria in Uganda and Mozambique.

## Methods/Design

### Study aim and objectives

The aim of this study is to develop and evaluate innovative approaches for increasing CHW supervision, motivation, performance and retention, and assess the impact of these interventions on coverage of appropriate treatment for children with diarrhoea, pneumonia and malaria in Uganda and Mozambique.

### Primary objectives

#### Formative phase

The primary objectives during the formative phase of this study are as follows:

1. To identify innovative solutions with the potential to increase coverage of iCCM and improve its quality through better CHW performance, motivation and retention.
2. To assess the feasibility of these innovative solutions and assess their acceptability among community members, CHWs, facility-based health workers, sub-national and national health authorities.

#### Evaluation phase

The primary objectives during the evaluation phase of this study are as follows:

1. To evaluate the impact of the selected interventions on CHW performance, motivation, retention, and coverage of appropriate treatment for children with diarrhoea, pneumonia and malaria.
2. To assess the costs of the interventions and investigate their potential cost-effectiveness.

#### Dissemination phase

The primary objective during the dissemination phase of this study is as follows:

1. To promote the implementation and spread of iCCM by sharing with the Ministry of Health (MoH), sub-national health authorities and stakeholders the experiences and findings that improve coverage and quality of iCCM.

### Study context

The inSCALE study is collaboration among Malaria Consortium, the London School of Hygiene & Tropical Medicine, the Institute of Global Health at University College London, Karolinska Institutet, Makerere University College of Health Science and the Ministries of Health (MoH) in Uganda and Mozambique. inSCALE has been implemented in the Midwestern region of Uganda and in Inhambane Province in Mozambique. Both Uganda and Mozambique have a long history of implementing community case management for sick children.

Uganda was one of the first countries to scale up home management of Malaria from 2002 to 2006, introducing pre-packed chloroquine-sulphadoxine/pyrimethamine for presumptive treatment of fever by volunteer CHWs. In 2004, after the rebel war, UNICEF supported the MoH to pilot the implementation of home-based care of children with diarrhoea, pneumonia and malaria in Northern Uganda using oral rehydration solution (ORS), cotrimoxazole and artemether-lumefantrine (Coartem; Novartis). Beginning in 2006, the MoH instituted the Village Health Team (VHT) strategy to strengthen community capacity for health promotion and health service delivery. A VHT has 4 to 5 volunteers (referred to as ‘VHTs’) selected by the community and equipped with basic materials (storage boxes, badges, T-shirts and gumboots). In 2010, Uganda launched iCCM, whereby two community-elected VHTs per village were trained to manage children 2 to 59 months with malaria, pneumonia, and diarrhoea using diagnostics (malaria rapid diagnostic tests (mRDTs) and respiratory timers for detection of fast breathing) and treatment (dispersible artemether-lumefantrine, dispersible amoxicillin, ORS, zinc and rectal artesunate). These VHTs also carry out home visits for active case detection and referral of sick newborns. Children with severe malaria and pneumonia are given pre-referral treatment with rectal artesunate and amoxicillin, respectively. The VHTs are supervised by health workers from the nearest health facility who conduct quarterly supervision group meetings at the health facility, at which time the VHTs also replenish their medicine stocks. A transport refund of about US$ 4 is given to VHTs at these meetings. Health workers supplement group meetings at health facilities with monthly community visits to VHTs during the first 3 months after training [10] and subsequently on at least an annual basis.

In Mozambique, the MoH introduced the concept of the lay CHW through the ‘Agente Polivalente Elementar’ (APE) in the 1970s to support public health activities at the district level [11]. The programme, which was initially very strong despite implementation challenges, weakened in the 1980s due to a shift in focus in the health sector: from provision of primary health care at the community level to provision at the facility level, partly influenced by system breakdowns during the civil war. Some of the original APEs still continued to work, but a lack of supervision, re-training and effective integration into the health system resulted in low programme effectiveness. In 2007, the MoH held a national level meeting to refocus the roles and responsibilities of the APEs, and in 2010 the Minister endorsed the APE operational plan and revised curriculum [12]. Recommendations highlighted that APEs should be respected and trusted individuals selected by their community and trained by the MoH to promote better health within their community and provide preventive and curative care. They receive Kit C - a basic medicine kit including ORS and zinc for diarrhoea, amoxicillin for pneumonia, artemether-lumefantrine for uncomplicated malaria, and pre-referral rectal artesunate for children with severe malaria. In 2013, mRDTs and adult artemether-lumefantrine were also provided in a separate kit from the National Malaria Control Programme. The APEs diagnose, refer, and treat children between 2 and 59 months of age for diarrhoea and pneumonia, as well as all age groups with confirmed malaria. Guidelines recommended that the majority of their time (80%) should be spent in the communities doing house-to-house visits, holding health education talks (‘palestras’), and conducting active case detection and referral of sick newborns and pregnant women. As per the APE programme guideline, the APEs receive quarterly supervision from the nearest health facility (when medicine kits are replenished) in addition to six-monthly supervision by outreach teams from the district. APEs receive a monthly allowance of 60% of the minimum salary level (US$ 40).

### Study sites

#### inSCALE Uganda

**Figure 1:**
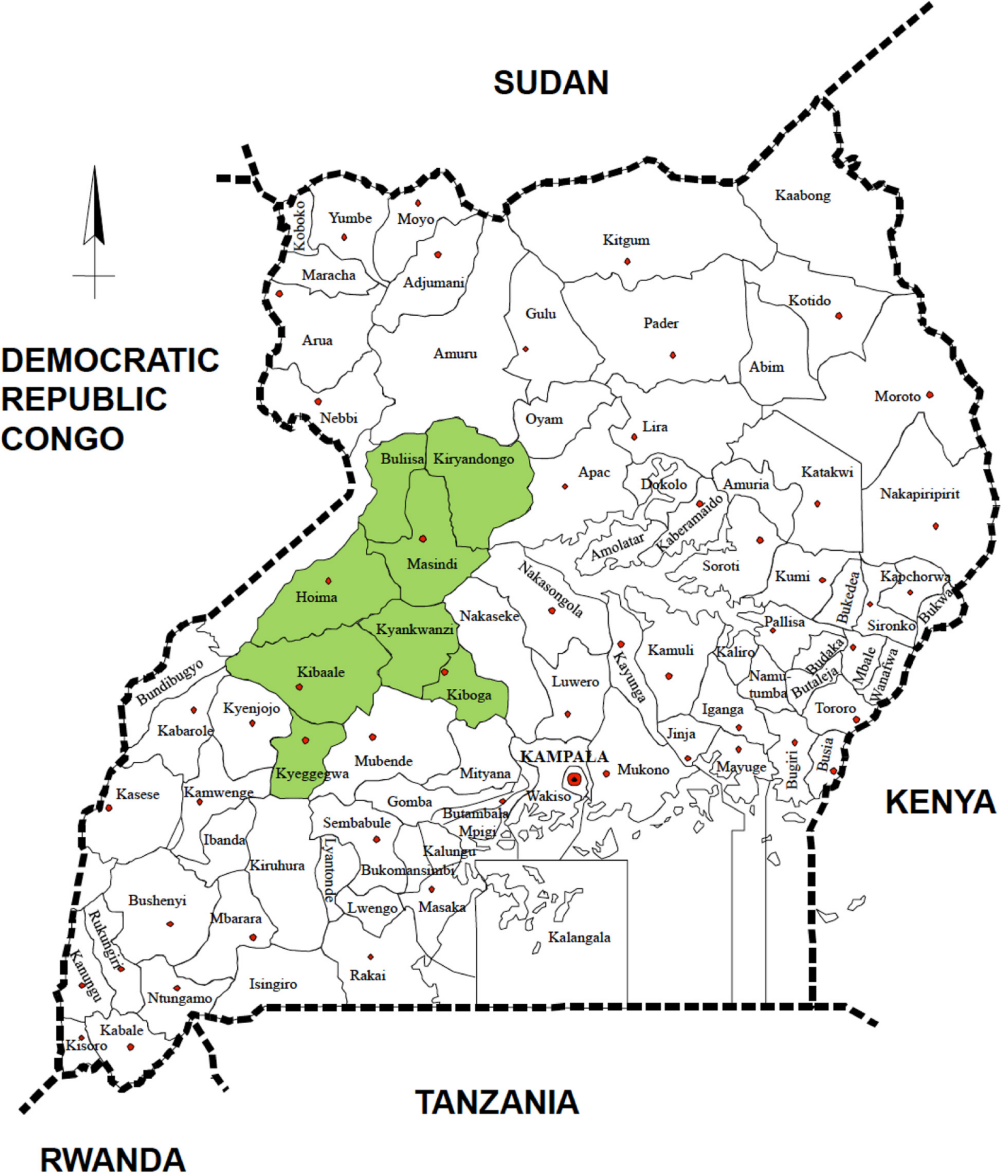
**inSCALE implementation area in Midwestern Uganda.**

inSCALE operates in eight districts in Midwestern Uganda (Buliisa, Masindi, Kibaale, Kyegegwa, Kyankwazi, Kiryandongo, Kiboga and Hoima), where iCCM implementation has been supported by the Malaria Consortium through a grant from the Canadian International Development Agency (CIDA) since August 2010 (Figure 1). Under the CIDA grant, Malaria Consortium provides technical support to the programme design; trains Master and district trainers; facilitates the training of VHTs; supplies health facilities and VHTs with medicines; and supports health facilities and districts to do monitoring and evaluation as well as supportive supervision. The districts where the projects have been implemented have an estimated 1.8 million people living in approximately 4,000 villages, with 20% being children under 5 years of age. The population is multiethnic and has a variety of cultural practices, with the majority of the population being able to read and write. It includes nomadic cattle herders, fishing communities, and peasant farmers. In the area, approximately 10,000 VHTs are operating, with 5,700 VHTs (approximately two per village or 1 per 250 people on average) trained in iCCM for 6 days between July 2010-June 2011. Each VHT sees, on average, 14 children every month [13].

#### inSCALE Mozambique

**Figure 2:**
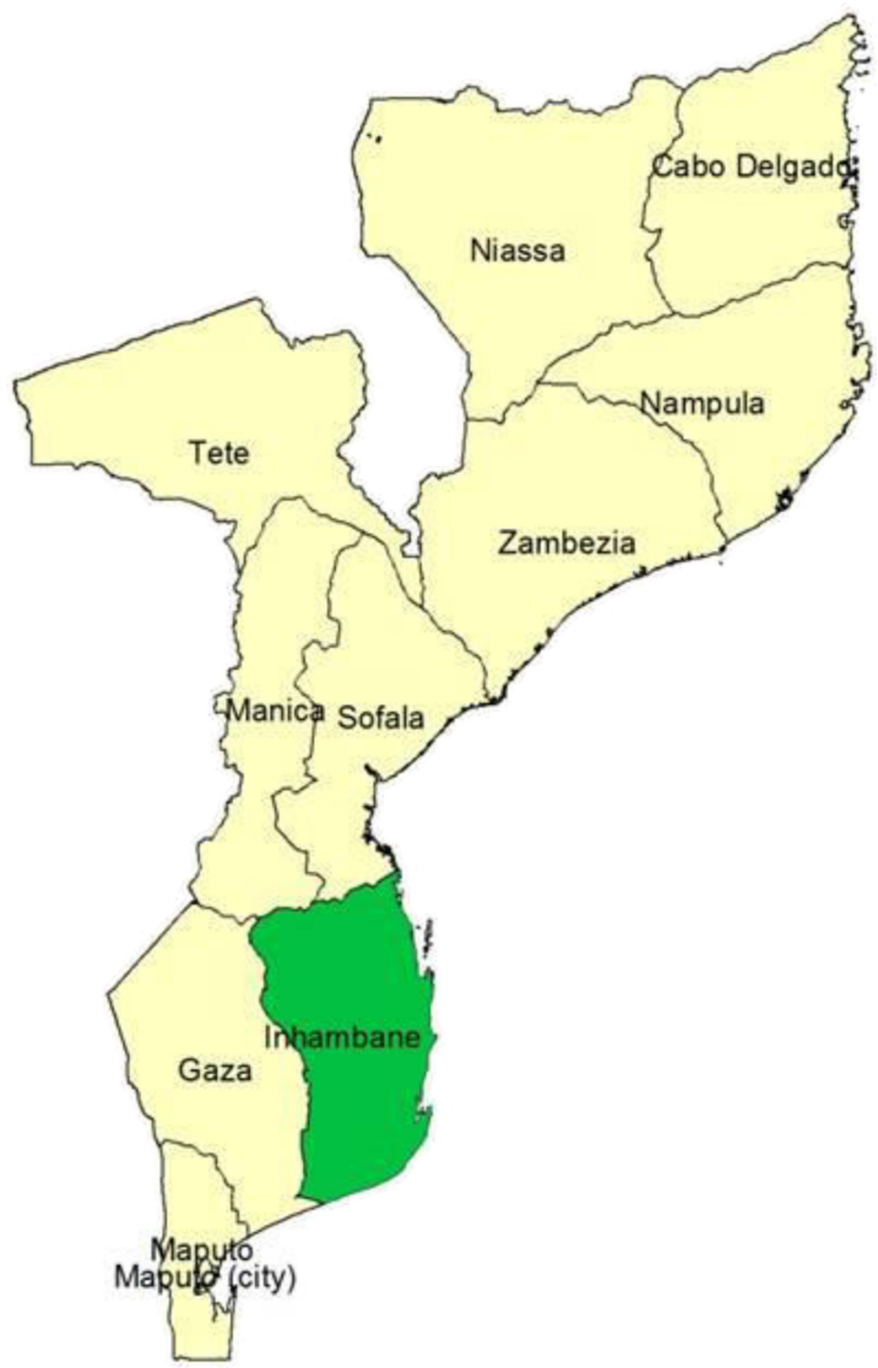
**inSCALE implementation area in Mozambique.**

inSCALE operates in all 12 districts in Inhambane Province (Funhalouro, Govuro, Homoíne, Inharrime, Inhassoro, Jangamo, Mabote, Massinga, Morrumbene, Panda, Vilankulo, and Zavala). Since 2011, iCCM implementation in seven of these districts was supported by Malaria Consortium, while UNICEF supported the other five (Figure 2). Under the same CIDA grant as for Uganda, the Malaria Consortium supported the revisions of the training manuals and job aids for the APEs; the implementation of the 4 months training; the provision of APE medicines to the provincial warehouse; and the implementation of supervision and monitoring and evaluation activities. The province and districts where the programmes were implemented have an estimated 1.3 million people living in approximately 145 villages (bairros), with 18% being children under 5 years of age. There are three ethnic groups whose constituents communicate mostly in Portuguese (the official language in Mozambique), Chope (the southern part of Inhambane) and Matsua (the northern part of Inhambane). The majority of the population are subsistence farmers with little or no literacy skills. In the province, 275 APEs were trained between August and December 2011 in the new curriculum, which includes a module of iCCM. Each APE serves approximately 500 to 2,000 people and sees, on average, 50 patients every month.

### The inSCALE interventions

inSCALE developed two integrated intervention packages based on extensive formative research [14-16]. At the beginning of the project, several reviews and consultations took place to ensure that the interventions designed drew on experience from previous work and appropriate theory. There was an additional focus on using these sources to identify areas of legitimate need with genuine potential for innovation. An initial team meeting determined what to review with three strands of enquiry identified and described below, and tasks were allocated to team members with relevant expertise, written up in reports [17] and presented and discussed in subsequent meetings:

1. Literature reviews of the areas of supervision, motivation theory, incentives, data use in quality improvement, mobile health (mHealth), business management and human resources, agriculture and community development.
2. Reviews of the history of Uganda and Mozambique as it relates to CHW programmes.
3. Consultations with stakeholders, academic and programme implementers working with health-focused CHWs in a variety of contexts to identify strategies that could increase CHW motivation and retention.

These reviews were used to inform the development of a conceptual framework on CHW motivation to perform and remain in role and to develop a list of potential alternative solutions to these problems - called ‘innovations’. Using the reviews and the theoretical findings, the inSCALE team selected a ‘long list’ of potential innovations to test based on ratings for a) impact potential, b) ability to fulfil required needs (in relation to CHW motivation and performance), c) acceptability, d) feasibility and e) sustainability. The long list of innovations fell into two clusters: a mHealth arm and a participatory community engagement arm. Both of these approaches aimed to positively influence CHW motivation, retention and performance by promoting their sense of collective identity.

**Figure 3:**
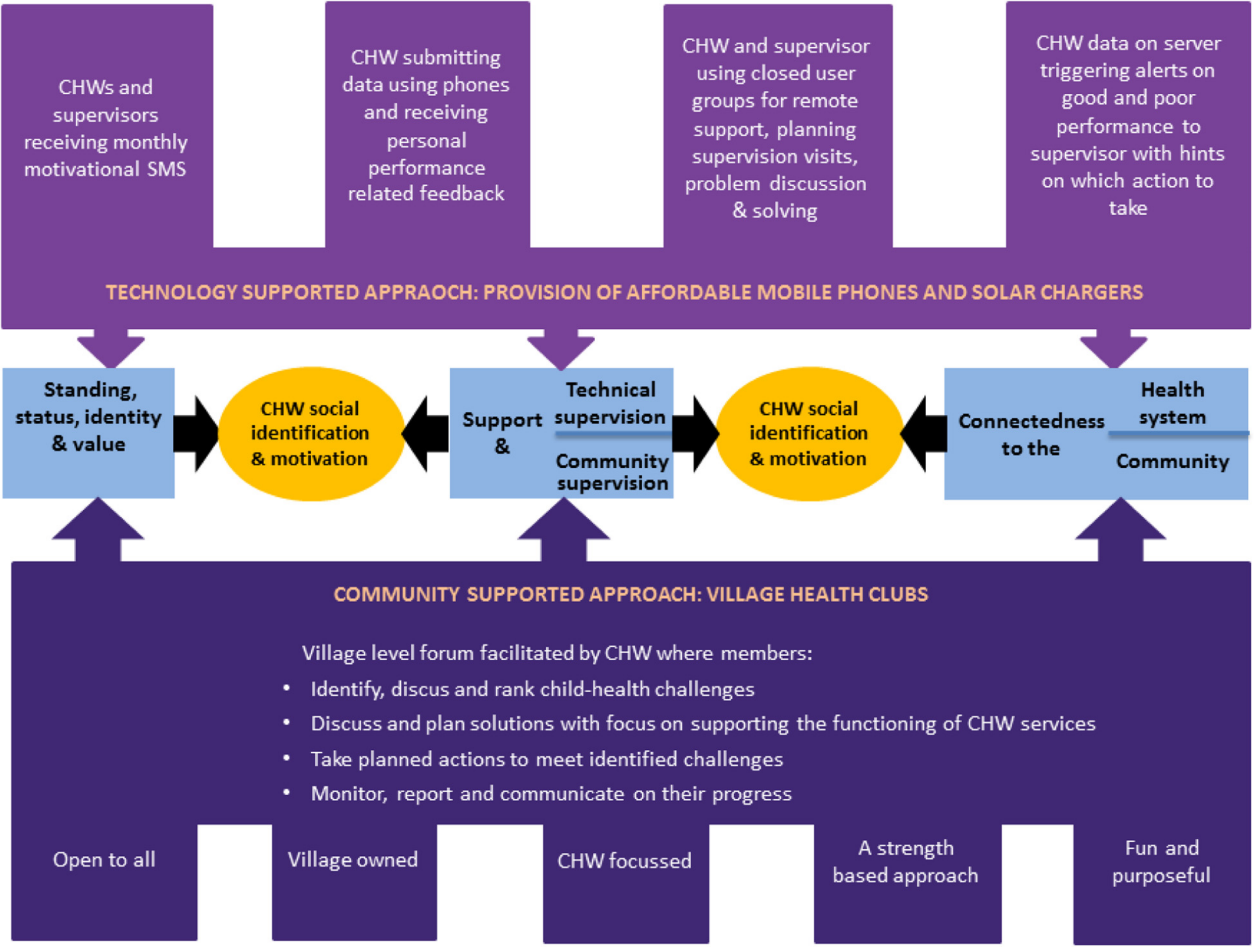
**Details of inSCALE’s two integrated intervention packages.**

The long list of potential innovations was presented to key personnel at the MoH in each country who gave their feedback on feasibility, acceptability and impact using pile sorting methodology [18], the results of which were used to eliminate several innovations felt to be unfeasible or unacceptable. Consequently, a list was created of 15 potential activities identified across the two intervention packages in the two countries. Formative research, gauging views of CHWs, their supervisors, district officials and key programme implementers, as well as caregivers, heads of households and traditional community leaders, was then conducted to ensure that innovations were acceptable and feasible at the community level, filled a need, and to determine the details of the approach (Strachan DL, Källander K, Nakirunda M, Ndima S, Muiambo A, Hill Z for the inSCALE study group, unpublished paper). Data were also collected on the current work of the CHWs and support that they received in order to understand motivation and retention and to identify priority issues. The results were used to finalise the two intervention packages. These consisted of a number of innovations (Figure 3) with both intervention packages implemented in Uganda and one in Mozambique [15]. Materials developed to support these packages were extensively reviewed and pre-tested.

#### Intervention package 1 - The participatory community engagement approach

In Uganda, the inSCALE project is supporting the implementation of an innovative participatory community engagement approach called the Village Health Clubs (VHCs). The VHC aims to improve child health through a community-led forum with the CHWs as the main focus point. VHC meetings are intended to provide a forum where CHWs and community members who are part of the club can work together to identify child health and CHWs challenges. They use village networks, knowledge, creativity and other community assets to help solve child health problems. Village health clubs are implemented through a four-step learning, planning and action cycle facilitated by the CHW. The time between each step is 1, 2 or 4 weeks depending on the decision taken by the club members. Health club members rank child health challenges faced by their community using picture cards. They discuss solutions, which include supporting the functioning of CHW services, and take actions to meet these challenges. Throughout the cycle the health clubs monitor and report on their progress.

Village health clubs are open to all members of the village and designed to be fun while focusing on the CHW as the main village health asset. A participatory empowerment approach is adopted where the CHW (as facilitator) encourages members to plan and carry out the club’s activities. They also promote group decision-making and ownership. Solutions to village health challenges developed by village members are a key focus of the village health club approach. Table 1 describes the contents of the participatory community engagement intervention in Uganda.

**Table 1:** **Content of the participatory community engagement intervention in Uganda**

**Intervention components**	**Uganda**
Key principles	Village health clubs (VHCs) aiming to improve child health through a community led-forum with the village health team (VHT) as the main focus point.
	Based on five main pillars:
	• open to all
	• village owned
	• intended to support VHT work
	• strength based (using village assets), and
	• fun and focused
Training and club facilitation	Two VHT club facilitators from each village trained for four days to encourage club members to plan and carry out the club’s activities using an action and planning cycle.
Accessories and materials	• Picture cards for ranking common child health problems
	Instructional VHC flip books
	• T-shirts
	• Membership cards
	• Stamps and other stationary to help with the establishment and operation of the clubs in the communities
Supervisor support and patrons	Village leaders were appointed as patrons and sensitised to support the mobilisation of the communities to join the clubs.
	VHT supervisors, health assistants and sub-county development officers were trained by District Health Educators and Malaria Consortium master trainers in effective supervision skills using a core competency assessment tool, and as trainers of VHTs in the VHC intervention.
Numbers of users	A total of 880 VHTs across the eight districts, to facilitate the set-up of 440 VHCs.

#### Intervention package 2 - the mHealth supported approach

In Mozambique and Uganda, inSCALE is giving CHWs phones with which they can send their weekly reports, receive immediate automated feedback on performance and access a closed user group with their supervisors in order to increase communication and support. Every month, a motivational performance-related SMS is sent out, and supervisors receive weekly automated actionable messages for CHWs who are performing at high or low standards. Table 2 describes the components of the mHealth intervention arm for Mozambique and Uganda.

**Table 2:** **Content of the mHealth intervention in Mozambique and Uganda**

**Intervention components (‘innovations’)**	**Mozambique**	**Uganda**
Mobile phone	Samsung Galaxy Y (Android smart phone)	Nokia C2-00 (Java enabled dual SIM card feature phone)
Accessories	Solar lamp (Sun King Pro) with multiple phone charging pins	Solar lamp (Sun King Pro) with multiple phone charging pins
Software	‘InSCALE APE CommCare app’ providing audio and images for each step in the sick child assessment process. Individual patient data is synchronised with an online database, and aggregated patient data and drug stock reports are submitted on a weekly basis.	‘inSCALE Mobile VHT system’ to send aggregated weekly reports on patients seen (sex, mRDT results, symptoms and classification of signs, treatment given and outcome of treatment) and current drug stock levels.
		Respiratory rate application where user presses the centre button for each breath observed during one minute added to the phone tool box.
	Respiratory rate application where user taps the screen for each breath observed during one minute built into the electronic algorithm.	
Feedback messages	Off-line decision support for diagnosis, treatment and referral provided at the end of the consultation process.	Relevant and personalised feedback messages based on submitted data sent instantly after reports are received.
Supervisor support	Automated weekly and monthly reports are emailed to health facility and district supervisors on APE activities, suggesting targeted follow-up actions.	Automated SMS sent to supervisors flagging problems and strengths identified in the data submitted, and alerting supervisors about VHTs requiring targeted supervision.
		Trained as trainers and in effective supervision skills using paper based core competency assessment tools
	Supervisors’ phones programmed with an electronic checklist related to core APE competencies linked to an APE Performance Checklist.	

#### Supportive activities

Several supportive actions took place to promote the interventions before their introduction in the communities. In both countries, consensus dialogue meetings on the planned interventions, the randomisation process and the evaluation of the RCT were organised with local leaders, using printed sensitisation briefs and presentations. In Uganda, meetings were held with district and sub-county leaders, and in Mozambique, two sensitisation meetings were held with provincial and district leaders. After these meetings, the districts and sub county leaderships (Uganda) and provincial and district stakeholders (Mozambique) pledged their support towards the interventions and approved the forthcoming randomisation process.

#### Effect pathways of inSCALE’s interventions

**Figure 4:**
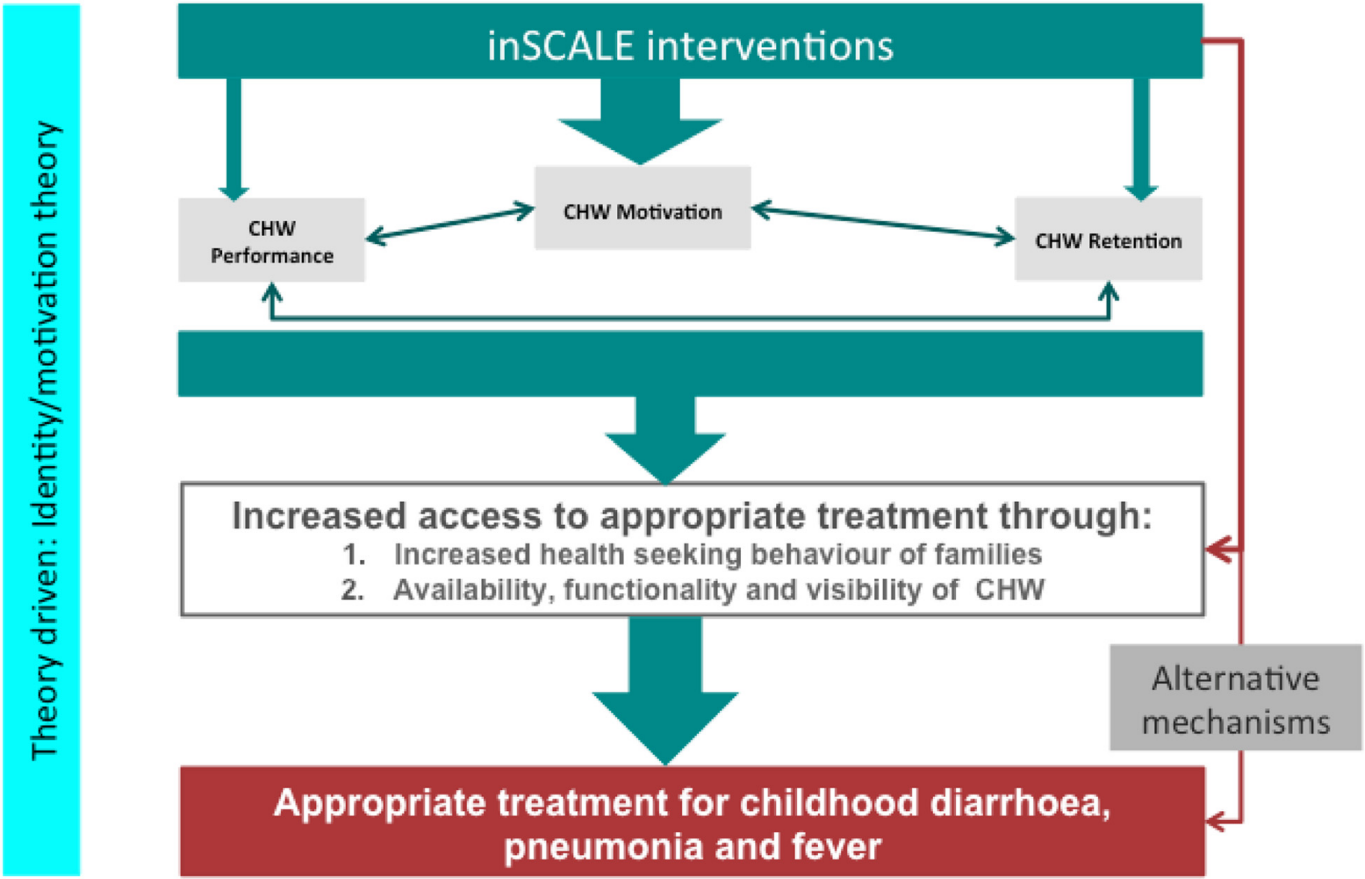
**inSCALE conceptual framework.**

Figure 4 outlines the conceptual framework for the evaluation of the inSCALE interventions and how they link to the main outcomes. Both interventions assume that motivation is the key to performance and retention, and that when these improve, the result will be improved access to services and, ultimately, improvements in appropriate treatment of sick children. The mHealth supported approach, using mobile phones with unlimited talk time and custom-made applications, is intended to improve the CHWs’ sense of connectedness to the health system and to their CHW peers. As a result of the increased support and performance-related feedback, it is hoped that the CHWs will feel an improved sense of status. The free talk time is expected to enable supervisors to provide targeted supervision, while the reminder messages and respiratory timers are meant to improve performance in diagnosis, treatment and referral. In Mozambique, the CHW performance was explored during the formative phase and was found to be lower than in Uganda, presumably because of the lack of effective job aids. Therefore, additional multimedia phone software was developed for Mozambique, which provides the CHW with a step-by-step guide to the consultation steps as well as treatment guidance when all steps are complete.

The community engagement approach aims to enhance the perceived value of the CHW, both for themselves and for the communities they serve, through inclusive and participatory local activities. The actions of health club members will help communicate to the CHW and village members that their work is important, of value and appreciated. In doing so, village health clubs (VHCs) will improve the status and standing of CHWs as key village health assets, increasing their motivation and the quality of service provision. VHCs will also increase the community’s understanding of what CHWs can and cannot do and their potential to improve child health in the village. This increased understanding could mean that a greater demand for CHW services and, correspondingly, an increase in the number of children accessing them. The training of CHWs to facilitate the health clubs will reinforce and add to skills acquired during iCCM training and may also improve quality of care. VHCs will hopefully become valued forums, increasing the capacity of both the CHW and the village inhabitants to address health issues in their communities and ensure children are receiving timely, effective and appropriate treatment.

There is also a possibility that both interventions could lead to substantial engagement of community members with child health issues, over and beyond what could be reasonably attributed to their relationship with CHWs. In this scenario, improved care-seeking will not only be due to increased CHW motivation and retention, but also to enhanced community knowledge and action for child health. This process will be captured through the alternative mechanism outlined in Figure 4.

### Overview of trial design

The inSCALE interventions are being evaluated through cluster randomised controlled trials (cRCTs) in Uganda and Mozambique. The clusters are sub-counties in Uganda and districts in Mozambique, and correspond to the lowest administrative units where CHW services are coordinated, with an average of 60 VHTs and 18 to 25 APEs per cluster, respectively. In Uganda, there are 95 clusters in the eight study districts. In order to be eligible for randomisation, a cluster had to have CHWs trained in iCCM by 31 January 2011 (3 months before starting the baseline survey). Clusters that had less than ten villages or that were participating in another Malaria Consortium mobile phone pilot study were excluded. Overall 41/95 clusters were eligible for randomisation. In Uganda, the trial consists of a three-arm cRCT with 13 clusters in each arm; two clusters were not randomised and were designated as spare/back-up clusters in case of the need to drop one of the selected 39. In Mozambique, there are 12 clusters, allowing a two-arm cRCT.

The trial planning started in April 2011, and the inSCALE interventions were developed and fully implemented in the intervention areas by the end of 2012 in Uganda and by the end of June 2013 in Mozambique. Impact data on the main outcomes was collected in an end-line household survey after 12 months of implementation in Uganda (March-May 2014) and will be collected after 18 months of implementation in Mozambique (February 2015). Detailed process, cost and cost-effectiveness evaluations are being carried out in both countries. All data collection is expected to be completed in May 2015, and results will be available by the later part of 2015.

### Randomisation

Restricted randomisation was performed to minimise the difference between the intervention and control arms for key indicators of the average proportion of children appropriately treated for fever, diarrhoea, and pneumonia (FDP); VHT motivation (mean); and the mean (log10) cost of treatment for children with FDP [19]. The randomisation of clusters to the three arms was restricted to those randomisation schemes where there were minimal between-arm differences for these five key parameters but still allowed adequate numbers of randomisation schemes, as too few schemes could elicit selection bias [20].

In Mozambique, appropriate treatment for childhood FDP could not be measured reliably at baseline due to a delay in the roll-out of the APE kits containing iCCM medicines. Attempts to seek care from an APE or a public facility were chosen as proxy markers for overall appropriate treatment, as these showed the strongest correlation with appropriate treatment. This was based on an assessment of the relationship between appropriate treatment with these and other candidate variables using parallel data from the Uganda baseline survey (where appropriate treatment was measured), and by assessment of the cluster averages and variances for several care seeking variables from the Mozambique dataset itself.

**Figure 5:**
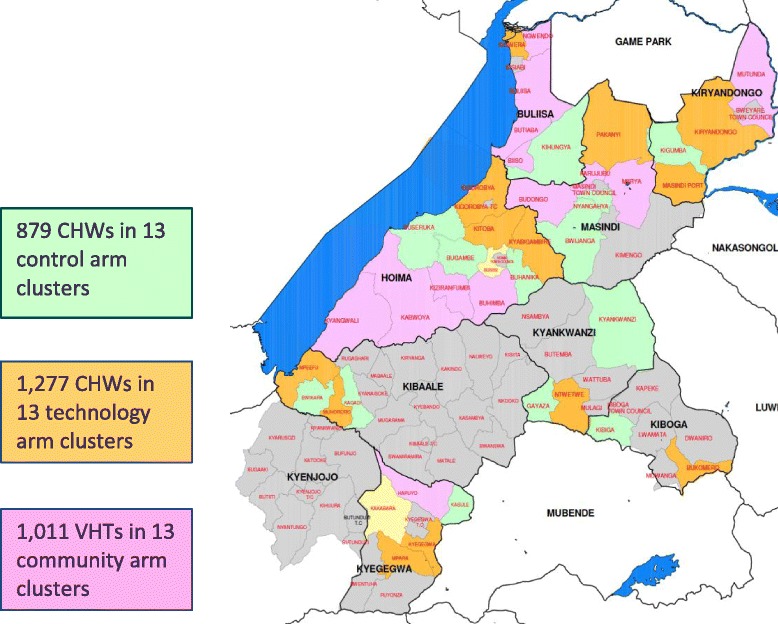
**Interventions and control arm clusters in Uganda.**

In Uganda, out of 500,000 random allocations of 39 clusters to three arms, 13,683 of the allocations fit all criteria. After applying a further filter to ensure that all districts had at least one sub-county in the mHealth intervention arm, and that at least the larger districts had a control arm, a final scheme was picked from the resulting sub-sample of 1,791 allocation schemes. In this scheme, all districts had at least one sub-county in the control arm and one in the mHealth intervention arm, and five out of eight districts also had at least one sub-county in the participatory community engagement arm (Figure 5).

**Figure 6:**
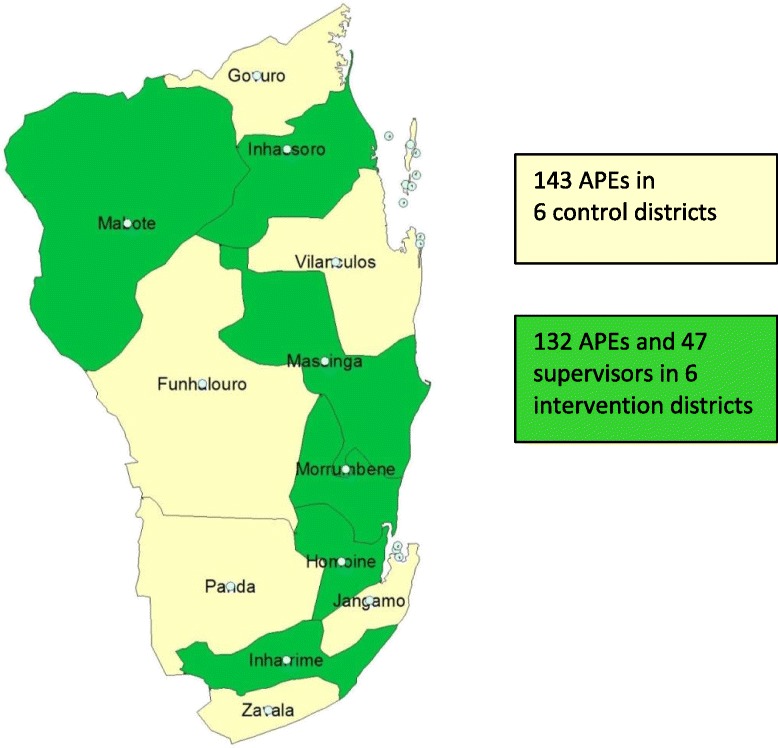
**Intervention and control arm clusters in Mozambique.**

Within Mozambique, there were 924 possible unique ways to sort 12 districts into two groups. In total, 84 schemes from these potential 924 met the all restriction criteria. A scheme was picked at random from the final 84 schemes, and was thus the final chosen scheme for the Mozambique study (Figure 6). In both sites, the selected schemes had no more than a 5% (proportions) or a 0.5 (scores/counts) difference between arms for any parameter. Sorting of clusters and random selection of schemes were carried out in Stata v12.1 (StataCorp, Texas USA).

### Intervention areas

The inSCALE interventions as described above were implemented in the 26 sub-counties in Uganda (13 in the ‘mHealth intervention arm’ and 13 in the ‘community engagement intervention arm’) and in six districts in Mozambique. All CHWs and children living in these clusters were potential recipients of the CHW intervention, in addition to having access to routine care currently available from private and public health services.

### Control areas

Children living in the control areas continued to benefit from the routine Ministry of Health iCCM package provided by the CHWs who were supported by the national and sub-national health services with funding from Malaria Consortium’s CIDA-iCCM project.

### Sample size

The sample size calculations, which are based on a cluster RCT with 13 and 6 clusters per arm in Uganda and Mozambique, respectively, are based on the main evaluation outcome: the percentage of children receiving appropriate treatment during illness episodes for each of pneumonia, diarrhoea and fever. This is also the outcome that requires the largest samples, as not all children surveyed will have had a recent illness episode. Inter-cluster coefficients of variation, baseline appropriate treatment rates, and prevalences of illness were calculated from data collected during the baseline surveys in each site and used in the sample size calculations (assuming a 5% significance level).

The calculation was based on the formula for comparison of proportions, adjusted for cluster effects stated in equation 7.7 of Hayes and Moulton [21], that is, where k is the between cluster coefficient of variation:

$$ C = 1 + \left(\mathrm{z}\upalpha /2 + \mathrm{z}\upbeta \right)2\left(\ \left(\uppi 0\left(1\ \hbox{--}\ \uppi 0\right)/\mathrm{m} + \uppi 1\left(1\ \hbox{--}\ \uppi 1\right)/\mathrm{m} + \mathrm{k}2\left(\ \uppi 02 + \uppi 12\right)\right)\ /\left(\uppi 0\ \hbox{--}\ \uppi 1\right)2\ \right) $$

The number of sick children required/cluster was determined by varying this number in the formula for the number of clusters until the number of clusters/arm equals 13 and 6, respectively [21]. Thus, sampling 155 children per cluster in Uganda would yield enough sick children to have 90% power to detect a 15% difference in the appropriate treatment of pneumonia and fever and a 20% difference in diarrhoea between arms. Sampling 390 children per cluster in Mozambique would yield enough sick children to have 90% power to detect 20% and 25% differences in appropriate treatment of fever and pneumonia respectively, and 80% power to detect a 25% difference in appropriate treatment of diarrhoea between arms.

The sample size formula and sampling scheme were also used to inform the design of the baseline surveys (which involved surveillance of households, CHWs and health facilities). At baseline, site-specific data was not available for k; therefore, a conservative estimate of 0.15 was used to inform the baseline sample size calculations in both sites. Prevalence values for FDP in the sites were based on estimates from recent Demographic and Health Surveys [22,23], Malaria Indicator Surveys [24,25], and in the case of Mozambique, an Integrated Health Systems and Child Friendly District Survey by UNICEF [26]. In Uganda, 3,900 households with children under the age of 5 years were sampled at baseline; of the 6,501 children in these households, 47% were reported as having had symptoms of fever, 11% with diarrhoea and 24% with pneumonia in the two weeks prior to the survey and were thus eligible for the full household survey questionnaire on treatment, care seeking, and health practices. In addition, 360 VHTs and the 79 health facilities supporting these VHTs were also sampled as part of the baseline. In Mozambique, 2,970 households with children under the age of 5 years were sampled; of the 4,422 children in these households, 29.2% were reported as having had symptoms of fever, 5.2% with diarrhoea and 10.7% with pneumonia in the two weeks prior to the survey. Data were also collected from 256 iCCM-trained APEs and the 80 health facilities to which they reported.

### Impact evaluation

The primary outcome is the change in carer reported appropriate treatment for fever, diarrhoea and pneumonia in children in households receiving the intervention(s) compared to those in areas with routine iCCM (‘control’ households). A proportion of children will have had two or more conditions simultaneously. We intend to group all conditions and assess the ‘use of appropriate drugs for this episode of illness for all illness episodes by intervention arm. Additionally, the effect of the interventions on appropriate treatment for each of the three conditions individually will be established as a way of understanding the pathways through which the interventions worked. The main comparison for effect will be obtained from the measured difference between interventions and control arms at end-line. Baseline characteristics were used to perform restricted randomisation to minimise the difference between the intervention and control arms on key indicators.

Secondary outcomes include a number of performance, retention, motivation, access and cost-effectiveness outcomes:

1. Mean motivation score (motivation outcome)
2. % CHWs staying in post after 1 year of implementation (retention outcome)
3. % monthly reports complete and submitted on time (performance outcome)
4. % CHWs with medicine stock-out <1 week each quarter (performance outcome)
5. % caregivers with sick child in the last 2 weeks who accessed iCCM treatment (coverage outcome)
6. % caregivers willing to use CHWs (access outcome)
7. % of start-up costs compared to recurring costs of the intervention packages (cost outcome)
8. Cost per CHW trained (cost outcome)
9. % of start-up costs compared to recurring costs of the intervention packages (cost outcome)
10. Cost per child who receives appropriate treatment for each of diarrhoea, pneumonia and fever (cost-effectiveness outcome)
11. Cost per episode of illness treated correctly (cost-effectiveness outcome)
12. Cost- per life year gained (LYG) and DALY averted (cost-effectiveness outcome)

#### Household, CHWs and health facility surveys

The primary outcome in both sites will be collected using an end-line household survey administered to the child’s primary carer. Data will be collected on illness prevalence, proportion of children who have received appropriate treatment and on household socio-economic status. Secondary outcomes, such as CHW performance, will be assessed in a sample of CHWs using a structured questionnaire with pre-tested case scenarios and knowledge questions administered by a trained research assistant. A motivation tool has been developed and validated to establish a composite index of CHW motivation. A health facility questionnaire will be used to collect data from health facility staff/supervisors on CHW supervision, support and health system costs.

The quantitative survey questionnaires will be adopted from the tools used for the baseline survey. These were developed in an iterative process: i) with the team of researchers in London and Kampala/Mozambique, ii) with potential field supervisors, and finally, iii) with trainees of the data collector training. Both the field supervisors and data collectors will visit local homes under supervision of a researcher to practice and test the questions and make revisions. All questionnaires will be written in English and translated into the local languages (Runyoro, Luo and Luganda for Uganda and Portuguese for Mozambique), and the accuracy of the translation will be checked by back translation into English. Key illness terminologies and other sensitive expressions will be translated into the native languages during the training and provided to the research assistants on a hard copy which guided them during the interview. Field editing will be done for consistency and completeness and after correction, data will be double-entered by trained data entry clerks into a SQL data management system capable of consistency, range, inter-database checks and audit trails.

#### Intention-to-treat analyses

All analyses will be intention-to-treat, and will account for the cluster-randomised design. Initial crude analyses will be based on summary measures for each outcome in each cluster and t-tests will be used to compare each intervention arm with the control arm. Multivariate random-effects models, which allow for cluster-specific constant amounts and work well for normally distributed outcomes and Poisson outcomes, will be used to assess the effect of the intervention on retention, motivation and coverage of appropriate treatment; adjustments will be made to account for clustering and any imbalances found between groups at baseline.. Random effects logistic regression models can be computationally demanding, and can fail to converge; if this is the case, generalized estimating equations (GEE) will be used instead if the random effects are found to be unreliable. The GEE approach modifies both parameter estimates and standard errors to allow for clustering and are appropriate where the clusters themselves are of no intrinsic interest. According to the analysis approach proposed [27], random effects models, whatever the outcome, will be used, only reverting to GEE where these do not converge.

#### Record review

During the project cycle, CHW record books will be reviewed to estimate the proportion of children classified with diarrhoea, malaria and pneumonia; the mRDT positivity rate, and frequency and outcome of referral. Drug stock cards will be reviewed to estimate drug stock-outs. Supervisor log books will be reviewed for frequency and content of supervision visits, and reports from district and health facility staff will be reviewed for CHW retention. Health facility records will be reviewed for patient load for health system costs. To estimate costs of CHW activities, the results of the process evaluation interviews described below will be combined with data collection from financial and activity reporting.

### Process evaluation and intervention monitoring

Process evaluation (PE), which started approximately 10 months after implementation of the interventions (January to February 2014 in Uganda and April to May 2014 in Mozambique), aims to identify lessons learned, successes and challenges and to crystallise best practices using key informant interviews with CHWs and their health facility supervisors. In Uganda, 24 VHTs and eight supervisors in the mHealth intervention, and 24 VHTs and eight supervisors in the participatory community engagement intervention in the districts of Buliisa, Hoima, Kyegegwa and Masindi were interviewed using predesigned topic guides. In Mozambique, 33 semi-structured key informant interviews with 24 APEs and nine supervisors were conducted in the districts of Inharrime, Morrumbene and Inhassoro of Inhambane Province. The qualitative most significant change methodology [28] was applied during the process evaluation as a way of monitoring changes observed as a result of the iCCM programme, and the interventions implemented, to document success stories from community members, CHWs and health workers. Specifically the process evaluation explored:

1. whether the interventions were delivered as designed and taken up and utilised as intended.
2. which the context-based factors had an impact on aspects of work motivation and satisfaction and the existing approaches to measuring motivation, instruments that could be adapted to the specific case of CHWs, best practices and new methods.
3. whether the interventions influenced motivation, retention, performance and/or other factors.
4. whether increased motivation impacted on performance and retention, and whether this lead to children receiving more appropriate treatment.
5. how the programme was implemented, how the programme operated, the services it delivered, and the functions it carried out.

### Cost and cost-effectiveness evaluation

The cost and cost-effectiveness analyses will take a societal perspective, assessing the economic impact for all parties affected by the interventions.

#### Intervention costs

The costs of the intervention packages will be estimated both from a financial and economic perspective. Costs will include direct project expenditure incurred by the implementing partner, the costs borne by the MoH at the national and local level during the implementation phase, the value of the work time contributed by the volunteering CHWs in Uganda, and any non-reimbursed out-of-pocket costs incurred by the CHWs in relation to their work. In order to allow generalisability of the findings to other contexts, cost will be categorised by principal inputs and activities, with quantities and values collected separately as far as possible.

#### Household and community costs

Direct (out-of-pocket) costs as well as indirect costs (value of lost production) for households in relation to care and treatment-seeking will be measured at the end of the intervention trials across trial arms. Direct costs will include transport costs, out of pocket payments to service providers, and any money spent on items complementary to treatment. Indirect costs will focus on the time spent caring for the sick child and seeking care. Costs will be broken down by socioeconomic status.

#### Health facility costs

It is likely that resource use in local health facilities will be affected by the introduction of a new level of healthcare worker; due to changes in the numbers of children seeking care at the health centres, of severe cases seen due to early treatment in the community, and of cases referred. The unit costs of treating the illnesses included in the iCCM package at different facility levels will be estimated, including staff time, drugs, diagnostics and other consumables and the use of equipment and facilities. The costs per episode of care in different facilities will be brought together with health seeking behaviour information from the household survey to assess the net difference in resources use.

#### Cost-effectiveness analysis

The cost-effectiveness analysis will assess the incremental cost and outcomes of the two intervention and control arms in Uganda, and between the intervention arm and control arm in Mozambique. Incremental costs will be derived from the cost analyses above, combining unit costs with data on service provision/ utilisation from the trial surveys in each arm. Outcome measures will be based in the principal outcome measure of the intervention trial, the incremental difference in the number of sick children appropriately treated during the trial period. This outcome will then be converted into life-years gained (LYGs) and disability- adjusted life years (DALYs) averted using standard methods.

### Informed consent

Individual informed consent is being obtained from all respondents throughout the projects’ data collection phases. Information sheets containing information about the study are provided (or in the case of phone interviews, e-mailed in advance) in local languages (Luganda, Luo, Runyoro/Rutoro or Portuguese (in Mozambique)). Respondents are given time to read the information sheet, and key points are summarised verbally and questions answered. Agreement to participate is indicated by signature or for phone interviews through verbal consent. The individual’s right to refuse consent or to stop the interview at any time after consent has been given is preserved; individuals are not required to provide explanation for such decisions. Stakeholders are asked whether they consent to the inclusion of verbatim quotes in reports and for their name to be included in the list of interviewed persons. Confidentiality of all data collected is maintained at all times, except where it relates to routine monitoring of performance of CHWs. All CHWs are identified by a unique ID number. No hazardous substances or medical procedures are used in this study.

### Trial monitoring

The project has a nine-person Technical Advisory Group (TAG) to facilitate dissemination and uptake of any findings within Uganda and Mozambique, as well as to provide technical support. Members include influential experts and national stakeholder representatives, WHO and UNICEF representatives and advisers with expertise in public health and community-based research, iCCM, health policy analysis, and international health policy development. It is also attended by the principle investigators and members of the inSCALE study group.

The Data Monitoring and Ethics Committee (DMEC) has five external members with expertise in health economy, child health, epidemiology, iCCM and randomised controlled trials, chosen to provide guidance to the trial in respect of study design, data analysis and study safety. The DMEC is responsible for reviewing and evaluating data with respect to the effectiveness of the supervision/motivation interventions. Both committees met at the start of the project to examine trial conduct and progress and to advise the inSCALE technical team, and will meet again in the final year of the project. The DMEC is not carrying out any interim analyses, as the inSCALE intervention does not involve any drugs or medical procedures and as the evaluation is based on outcomes occurring over a period of just one year.

### Ethical approval

The trial protocol was approved by Makerere University Institutional Review Board in Uganda, the Uganda National Council of Science and Technology (ref. HS 958), the Comité Nacional de Bioética para a Saúde in Mozambique (ref. 331/CNBS/12) and London School of Hygiene & Tropical Medicine Ethics Committee in the UK (ref. 5762). In addition, approval was obtained from the district authorities and local leaders in the communities where the study is being conducted. The study has been registered as a randomised controlled trial with http://www.clinicaltrials.gov (identifier NCT01972321).

## Discussion

For child health interventions like iCCM to have impact when implemented at scale, it is crucial that cost-effective strategies that improve performance and motivation of CHWs are identified and evaluated in a ‘real-life’ health systems context. The current, multi-country, cluster randomised controlled trial aims to meet this need by assessing two innovative approaches using mHealth and participatory community engagement to increase CHW support, supervision and motivation in Uganda and Mozambique. We hypothesise that both interventions can lead to improvements in coverage of appropriate treatment for children with diarrhoea, pneumonia and malaria (primary outcome) and CHW retention rate, motivation and performance (secondary outcomes).

This study’s strength is that, as well as drawing on theoretical perspectives and evidence of best practices, it applies a user-centred design to the innovations based on extensive formative research. In so doing the potential for innovations to be acceptable, feasible and scalable is increased by prioritising evidence generated from the operating context through exploration of needs as stated by intended users (CHWs and national stakeholders). The multidisciplinary research team is an additional strength that enables the application of robust methods for both qualitative and quantitative evaluation of the innovations, as well as high quality implementation. A further strength is the development of an economic model, parameterised for each country using data from iCCM implementation, the inSCALE intervention trial, and secondary data sources, to support policy decisions in the study countries.

The lack of an accurate measurement to estimate coverage of appropriate treatment of sick children is one weakness of the study. Coverage of maternal, neonatal and child health (MNCH) interventions are typically monitored through household surveys, which estimate treatment rates based on 2-week recall of illness symptoms by caregivers. However, these survey tools identify children with reported symptoms, and for illnesses like pneumonia where the true prevalence in community settings is low, this methodology does not provide an accurate denominator of cases for monitoring treatment rates [29]. Issues with recall of diagnostic results and treatments provided have also been documented [30,31]. While we are aware of these limitations, we attempted to increase the validity of our estimates by increasing the recall period to 4 weeks to increase the number of recent cases detected in the survey, and including an expanded list of symptoms indicative of pneumonia in order to increase the specificity of the pneumonia case definition. We also used drug charts to increase the validity of reported treatments. Another possible weakness of the study is the lack of control over contextual factors and other changes in the health system in which the innovations are implemented. While this was anticipated and attempts are made to access district and provincial operational plans and internal project reports, lack of data on factors that may mediate the effect of the interventions [32], such as drug stock-outs, health staff rotation, and concurrent programme implementation by other partners, leaves us unable to plot these influences with absolute confidence in their accuracy.

If evidence yields support for cost-effective innovations that can generate a highly motivated and well-performing workforce of CHWs, there is potential for sustainable coverage of high quality iCCM at scale and significant reductions in child morbidity and mortality.

### Public engagement and dissemination

The inSCALE study is designed to directly influence policy and practice, especially for government-led scale-up of iCCM in the two countries. Implementation has been carried out in close consultation with policy makers. In addition, study materials and early results will be made accessible on the project website (www.malariaconsortium.org/inscale) well before the final evaluation is made. There has been close documentation of all implementation steps, challenges, innovations and experiences. In addition, the study employs a rigorous economic modelling for iCCM implementation in the two countries, in order to provide directly support to the iCCM policy agenda.

Trial findings will be shared promptly with the local district health teams and local dissemination meetings with the study populations will be held. Policy briefs will be prepared and circulated nationally and internationally to relevant policy and donor organisations, and national workshop held to discuss the findings, lessons learnt concerning implementation and policy implications. Trial findings will also be disseminated in national and international scientific meetings, and peer-reviewed publications will be produced on the impact of the intervention on appropriate treatment of sick children, cost and cost-effectiveness of the interventions, impact on CHW motivation and performance, and process outcomes and lessons learned concerning the interventions evaluated.

Selected stories generated for the most significant change methodology will be disseminated to policy makers, implementers and the general public using audio-visual and written messages.

## Trial status

The implementation of the interventions in Uganda is completed and end-line survey data has been collected. Mozambique is continuing implementation until April 2015. Final evaluation results will be available April 2015 for Uganda and September 2015 for Mozambique.

## Abbreviations

ACT: Artemisinine-based combination treatment
APE: agentes polivalentes elementares
CHW: community health worker
CIDA: Canadian International Development Agency
DMEC: data monitoring and ethics committee
FPD: fever, pneumonia and diarrhoea
GEE: generalized estimating equations
iCCM: integrated community case management
inSCALE: Innovations at Scale for Community Access and Lasting Effects
mHealth: Mobile Health
MoH: Ministry of Health
mRDT: Malaria Rapid Diagnostic Test
ORS: Oral Rehydration Therapy
PE: Process Evaluation
RCT: randomised controlled trial
TAG: technical advisory group
UNICEF: United Nations Children’s Fund
VHC: Village Health Club
VHT: Village Health Team
WHO: World Health Organisation
